# Supplementary material for: Double-stranded RNA prevents and cures infection by rust fungi
Source: Commun Biol. 2023 Dec 6;6:1234. doi: 10.1038/s42003-023-05618-z (PMC10700371; doi:10.1038/s42003-023-05618-z)
Supplement: Supplementary file 2 — Supplementary Information [file 42003_2023_5618_MOESM2_ESM.pdf]

Supplementary Information

Supplementary Table 1. ***Effect sizes of disease coverage and plant health measures in control and treatment groups at all preventative and curative timepoints.*** Effect sizes are calculated according to Hedge's g, and effect sizes of 0.15, 0.40, and 0.75 are considered as small, medium, and large, respectively.

Disease Coverage								
Preventative			Curative 24 hr			Curative 6 dpi		
Group 1	Group 2	Effect size	Group 1	Group 2	Effect size	Group 1	Group 2	Effect size
Control	GFP	0.39	Control	GFP	0.23	Control	GFP	0.10
Control	BTUB	3.38	Control	BTUB	2.64	Control	BTUB	4.12
Control	EF1-A	3.81	Control	EF1-A	2.38	Control	EF1-A	3.29
GFP	BTUB	3.44	GFP	BTUB	2.90	GFP	BTUB	2.98
GFP	EF1-A	3.44	GFP	EF1-A	2.65	GFP	EF1-A	2.48
BTUB	EF1-A	0.21	BTUB	EF1-A	0.12	BTUB	EF1-A	0.86
**Plant Health**								
Curative 24 hr			Curative 6 dpi			Curative 14 dpi		
Group 1	Group2	Effect size	Group 1	Group 2	Effect size	Group 1	Group 2	Effect size
Control	GFP	0.19	Control	GFP	0.13	Control	GFP	0.51
Control	BTUB	1.84	Control	BTUB	2.81	Control	BTUB	1.68
Control	EF1-A	2.21	Control	EF1-A	1.78	Control	EF1-A	2.54
GFP	BTUB	1.64	GFP	BTUB	2.27	GFP	BTUB	1.55
GFP	EF1-A	2.02	GFP	EF1-A	1.55	GFP	EF1-A	2.46
BTUB	EF1-A	0.40	BTUB	EF1-A	0.56	BTUB	EF1-A	0.33

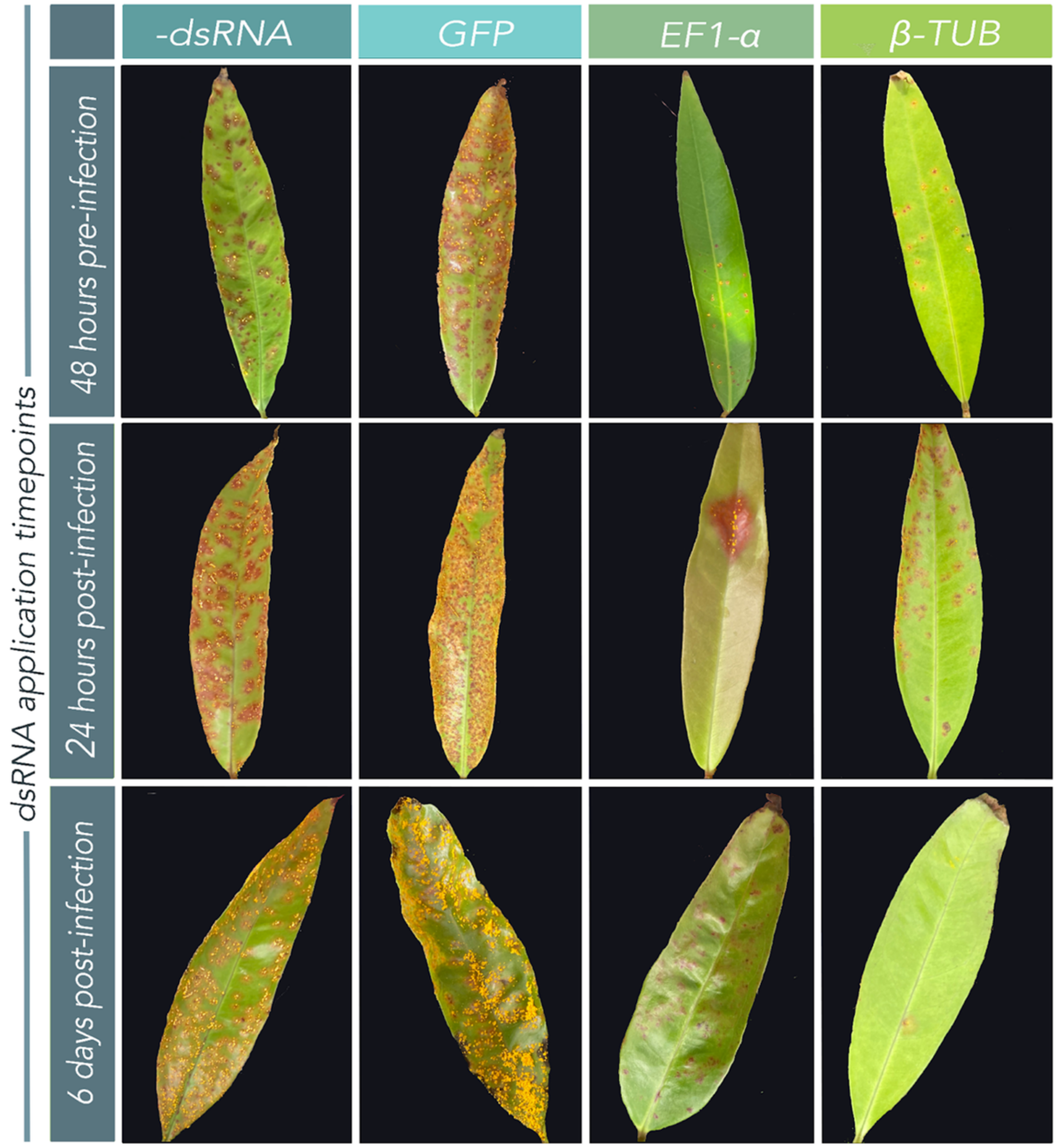

Supplementary Figure 1. ***Representative leaves used for disease assessments in the Leaf Doctor application from* Syzygium jambos *plants infected with* Austropuccinia psidii *at all preventative and curative timepoints.*** *Syzygium jambos* trees (n=6) were infected with *A. psidii* urediniospore inocula and treated with a nuclease free H_20 (negative control)), a non-specific dsRNA control (*GFP* dsRNA), or *beta- tubulin (β-TUB)*, or *transcription elongation factor (EF1-a) A. psidii*-specific dsRNAs at a concentration of 100 ng/μL at 48 hours pre-infection, 24 hours, or 6 days, post-infection. Each leaf is representative of technical and biological replicates in its treatment or control group. In the Leaf Doctor application, areas that are yellow, orange, pink, or red in colour will be scanned as 'diseased' and areas that are various shades of green will be scanned as 'healthy', allowing the application to determine a percent (%) disease coverage of leaf.
