# Supplementary material for: Double-stranded RNA prevents and cures infection by rust fungi
Source: Commun Biol. 2023 Dec 6;6:1234. doi: 10.1038/s42003-023-05618-z (PMC10700371; doi:10.1038/s42003-023-05618-z)
Supplement: Supplementary file 3 — Reporting Summary [file 42003_2023_5618_MOESM3_ESM.pdf]

nature portfolio

Corresponding author(s): Miss Rebecca Degnan & Dr Anne Sawyer

Last updated by author(s): 03/11/2023

Reporting Summary

Nature Portfolio wishes to improve the reproducibility of the work that we publish. This form provides structure for consistency and transparency in reporting. For further information on Nature Portfolio policies, see our Editorial Policies and the Editorial Policy Checklist.

Statistics

For all statistical analyses, confirm that the following items are present in the figure legend, table legend, main text, or Methods section.

n/a	Confirmed	
☐	☒	The exact sample size (*n*) for each experimental group/condition, given as a discrete number and unit of measurement
☐	☒	A statement on whether measurements were taken from distinct samples or whether the same sample was measured repeatedly
☐	☒	The statistical test(s) used AND whether they are one- or two-sided<br>*Only common tests should be described solely by name; describe more complex techniques in the Methods section.*
☐	☒	A description of all covariates tested
☐	☒	A description of any assumptions or corrections, such as tests of normality and adjustment for multiple comparisons
☐	☒	A full description of the statistical parameters including central tendency (e.g. means) or other basic estimates (e.g. regression coefficient) AND variation (e.g. standard deviation) or associated estimates of uncertainty (e.g. confidence intervals)
☐	☒	For null hypothesis testing, the test statistic (e.g. *F*, *t*, *r*) with confidence intervals, effect sizes, degrees of freedom and *P* value noted<br>*Give P values as exact values whenever suitable.*
☒	☐	For Bayesian analysis, information on the choice of priors and Markov chain Monte Carlo settings
☒	☐	For hierarchical and complex designs, identification of the appropriate level for tests and full reporting of outcomes
☐	☒	Estimates of effect sizes (e.g. Cohen's *d*, Pearson's *r*), indicating how they were calculated

Our web collection on statistics for biologists contains articles on many of the points above.

Software and code

Policy information about availability of computer code

Data collection	Disease coverage data were collected using the Leaf Doctor mobile application.
Data analysis	Welch’s two sample, two-tailed t tests were computed in R Studio 1.3.109335 at a 95 percent confidence interval using the dplyr package and pairwise t-test function52. p values of <0.05 were considered significant and >0.05 were considered not significant. Data were plotted in R Studio 1.3.109335 using box (geom_box) and scatter (geom_scatter) plots with ggplot2 package52. Hedge’s g was used to quantify to magnitude of difference, or effect size, between control and/ or treatment groups. Hedge’s g was computed in R Studio 1.2.1093.

For manuscripts utilizing custom algorithms or software that are central to the research but not yet described in published literature, software must be made available to editors and reviewers. We strongly encourage code deposition in a community repository (e.g. GitHub). See the Nature Portfolio guidelines for submitting code & software for further information.

Data

Policy information about availability of data

All manuscripts must include a data availability statement. This statement should provide the following information, where applicable:

- Accession codes, unique identifiers, or web links for publicly available datasets
- A description of any restrictions on data availability
- For clinical datasets or third party data, please ensure that the statement adheres to our policy

Raw disease coverage scores, plant health ratings, Fv/Fm and gsw data, and all TPEF and SEM micrographs that support the findings of this study are available on request to the authors, and will be made publicly available via Dryad upon publication.

Research involving human participants, their data, or biological material

Policy information about studies with human participants or human data. See also policy information about sex, gender (identity/presentation), and sexual orientation and race, ethnicity and racism.

Reporting on sex and gender	N/A
Reporting on race, ethnicity, or other socially relevant groupings	N/A
Population characteristics	N/A
Recruitment	N/A
Ethics oversight	N/A

Note that full information on the approval of the study protocol must also be provided in the manuscript.

Field-specific reporting

Please select the one below that is the best fit for your research. If you are not sure, read the appropriate sections before making your selection.

☒ Life sciences ☐ Behavioural & social sciences ☐ Ecological, evolutionary & environmental sciences

For a reference copy of the document with all sections, see nature.com/documents/nr-reporting-summary-flat.pdf

Life sciences study design

All studies must disclose on these points even when the disclosure is negative.

Sample size	No sample size calculations were performed. Sample sizes were determined based on availability of 1 to 2-year-old Syzygium jambos trees, which are relatively slow growing, as well as cost and time considerations relating to the synthesis of large volumes of dsRNA. Sample sizes were also decided based off similarity to sample sizes in previously published studies, and to ensure the adequate capture of biological variability.
Data exclusions	No data were excluded from the study.
Replication	This experiment was replicated successfully. Methods detailed in this manuscript also provide sufficient information for all experiments to be further reproduced.
Randomization	Trees were allocated to groups based roughly on age and size; considerations were taken to ensure that all control and treatment groups received an even distribution of larger and smaller trees.
Blinding	Investigators were not blinded. Blinding was not necessary as disease coverage assessments were automated using the Leaf Doctor application, and plant health assessments were made according to a strict rating scale, with no room for subjectivity. LI-COR measurements (Fv/Fm and gsw) were also made using the LI-600 315 Porometer/Fluorometer, and were not open to subjectivity.

Reporting for specific materials, systems and methods

We require information from authors about some types of materials, experimental systems and methods used in many studies. Here, indicate whether each material, system or method listed is relevant to your study. If you are not sure if a list item applies to your research, read the appropriate section before selecting a response.

Materials & experimental systems

n/a	Involved in the study
☒	☐ Antibodies
☒	☐ Eukaryotic cell lines
☒	☐ Palaeontology and archaeology
☒	☐ Animals and other organisms
☒	☐ Clinical data
☒	☐ Dual use research of concern
☐	☒ Plants

Methods

n/a	Involved in the study
☒	☐ ChIP-seq
☒	☐ Flow cytometry
☒	☐ MRI-based neuroimaging
